# Association between frailty and adverse outcomes after cardiac resynchronization therapy: a systematic review and meta-analysis

**DOI:** 10.1007/s41999-024-01112-9

**Published:** 2024-12-04

**Authors:** Xiaowang Li, Fei Fang

**Affiliations:** 1 Cardiovascular Interventional Treatment Center, First Affiliated Hospital of Huzhou University, Huzhou First People’s Hospital, Huzhou, 313000 Zhejiang China; 2 Geriatrics Department, Huzhou Third Municipal Hospital, The Affiliated Hospital of Huzhou University, 2088 Tiaoxi East Road, Wuxing District, Huzhou, 313000 Zhejiang China

**Keywords:** CRT, Frailty, Frail adults, Cardiac resynchronization therapy, Mortality, Response to CRT, Hospitalization, Readmission, Decompensated heart failure, Treatment response, Systematic review, Meta-analysis

## Abstract

**Aim:**

To synthesize evidence, using data from published studies, on the association of frailty with the outcomes after cardiac resynchronization therapy (CRT).

**Methods:**

The systematic search of PubMed, Web of Science, Scopus, and Embase databases was done to identify observational studies (cohort/case–control/cross-sectional) that used an objective method for frailty assessment and had presented adjusted effect sizes. STATA version 15.0 was used to conduct analysis, which was based on random effects model.

**Results:**

Fifteen studies were included. Frailty was found to be associated with an increased risk of in-hospital mortality (odds ratio (OR) 6.96, 95% confidence interval (CI) 5.48, 8.85). The effect of frailty on the response to CRT was not statistically significant (OR 0.55, 95% CI 0.19, 1.59). The pooled effect size indicated that frailty was associated with somewhat bigger but not statistically significant increase in the risk of complications (OR 1.70, 95% CI 0.93, 3.12). The risks of mortality and decompensated heart failure on long-term follow up were higher in frail patients (Hazard ratio (HR) 1.75, 95% CI 1.40, 2.17 and HR 3.03, 95% CI 1.33, 6.90, respectively) compared to patients without frailty. The risk of readmission was higher in frail patients, however, it did not achieve statistical significance (HR 2.63, 95% CI 0.89, 7.75).

**Conclusion:**

Frail CRT patients could be at higher risks of mortality, decompensated heart failure, and may have potentially higher rates of complications. Integrating frailty assessment into pre-CRT evaluation and customizing interventions for frail patients might be an essential steps towards enhancing outcomes in this population.

**Supplementary Information:**

The online version contains supplementary material available at 10.1007/s41999-024-01112-9.

## Key summary points

**Aim:**

To assess the impact of frailty on the outcomes such as risk of mortality, readmission, and decompensated heart failure in patients who undergo cardiac resynchronization therapy (CRT).

**Findings:**

Our results showed the association of frailty with the increase rates of adverse outcomes in patients after CRT.

**Message:**

It will advance our understanding of the complex interplay between frailty and CRT outcomes and inform evidence-based strategies to optimize care for this vulnerable population.

**Supplementary Information:**

The online version contains supplementary material available at 10.1007/s41999-024-01112-9.

## Introduction

Cardiac resynchronization therapy (CRT), also called as biventricular pacing, has emerged as an important intervention in managing heart failure, particularly in patients with reduced left ventricular ejection fraction and intraventricular conduction delay [1, 2]. It involves the implantation of a specialized pacemaker that coordinates the timing of contractions between the heart’s left and right ventricles [1, 2]. By restoring synchronized ventricular contraction, CRT can improve cardiac function, alleviate symptoms, and reduce the risk of heart failure-related complications. Despite its considerable benefits in improving cardiac function and symptoms, CRT is still associated with a substantial variability in the outcomes [3, 4]. Frailty, characterized by decreased physiologic reserves and increased vulnerability to stressors, has gained increasing attention for its potential impact on CRT outcomes. Numerous studies indicate that frailty is a significant determinant of health outcomes in older patients [5–7]. While frailty is traditionally associated with aging, its prevalence and relevance extend beyond chronological age, encompassing a complex interplay of physiological, psychological, and social factors [8, 9].

While the influence of frailty on the prognosis in patients with various cardiovascular conditions is well-documented, specific association with adverse outcomes following CRT is still unclear [10, 11]. Understanding this relationship is crucial, as frailty could potentially serve as a prognostic marker to identify patients at higher risk for poor CRT outcomes. Moreover, clarifying the impact of frailty on CRT efficacy and safety could inform tailored management strategies and improve patient selection for this intervention. To date, there is no systematic review of available studies on the association between frailty and outcomes after CRT. A previous narrative review by Chen et al. that included nine studies and attempted to present the findings on the effect of implantable cardioverter defibrillators in preventing sudden cardiac death in older adults [12] found higher rates of mortality in frail patients. However, the review included studies with substantial variability and lack of standard criteria in the categorization of frailty [12].

The current review aims to comprehensively synthesize the existing literature to provide contemporary evidence on the impact of objectively assessed “frailty” on CRT outcomes, such as mortality, readmission/hospitalization, and decompensated heart failure.

## Methods

### Criteria for selecting studies

The PRISMA guidelines were followed [13]. The Study protocol was registered at PROSPERO (registration number CRD42024537023). We considered studies that explored the association between pre-procedural frailty and adverse outcomes after CRT. For this review, CRT was considered as devices specifically designed for cardiac resynchronization, which includes both CRT with pacemaker capability (CRT-P) and CRT with defibrillator capability (CRT-D). Only studies where CRT meant use of devices for cardiac resynchronization, either with or without defibrillation (CRT-P or CRT-D) were considered.

#### Inclusion criteria

Eligible studies should have involved adult participants (≥ 18 years) and had provided objective and acceptable quantitative measures of frailty that was assessed prior to CRT. Given the nature of the study question, only observational studies (cohort/case–control/cross-sectional design) were included. The relevant outcomes of interest were mortality, hospitalization/readmission, and decompensated heart failure. Only studies providing adjusted effect sizes were included. This was deliberately done to exclude studies that reported association without adjustment for comorbidities, sociodemographic, biochemical and clinical parameters that could influence the association between frailty and outcomes reported. The studies published in peer-reviewed journals were included.

#### Exclusion criteria

We excluded studies involving permanent pacemakers or other types of cardiac devices not intended for resynchronization. The studies that based their frailty assessment solely on body mass index (considering underweight individuals as “frail”) or on basis of advancing age and presence of comorbid conditions or the inability to walk or mean daily activity performed were excluded. The studies solely focusing on technical aspects of CRT procedures, without evaluating clinical outcomes or frailty measures, were also excluded. Case-reports, conference abstracts and reviews were not considered for inclusion.

### Search strategy

Search was conducted using PubMed, Web of Science, Scopus, and Embase databases using specific keywords: (“frailty” OR “frail elderly” OR “frail older adults” OR “frail phenotype”) AND (“cardiac resynchronization therapy” OR “CRT” OR “biventricular pacing” OR “CRT-D” OR defibrillation OR pacemaker) AND (“adverse outcomes” OR “complications” OR “mortality” OR “hospitalization” OR CRT failure). The search scope was until March 31, 2024.

### Process of selecting studies

The search strategy was executed across the different databases. The initial pool of studies was deduplicated and two authors independently scrutinized the titles and abstracts of the remaining studies. The full texts of the relevant studies were assessed for eligibility. Data extraction from the finalized set of studies was independently carried out by the two authors using a structured form. All differences were resolved through discussions. The quality assessment was conducted using the Newcastle–Ottawa Scale (NOS), wherein each study had the potential to score a maximum of 9 points [14]. Scores surpassing 6 were deemed indicative of satisfactory quality.

### Statistical analysis

The analysis was conducted in STATA version 15.0 using the “metan” command. The pooled effect sizes were presented as odds ratio (OR) or hazard ratios (HR) with 95% confidence intervals (CI). A random-effects model was used for all outcomes to account for methodological differences and variations in the characteristics of the participants among the included studies [15]. Funnel plots and Egger’s test were used to assess for presence of publication bias [16]. Given the variability in how frailty was assessed across the included studies, a sensitivity analysis was conducted focusing on studies that employed a frailty index, a comparatively more homogeneous approach for assessment of frailty. The findings from this sensitivity analysis are presented for outcomes where a sufficient number of studies were available for pooling. A p-value below 0.05 was considered statistically significant.

## Results

### Selection of the studies

**Fig. 1:**
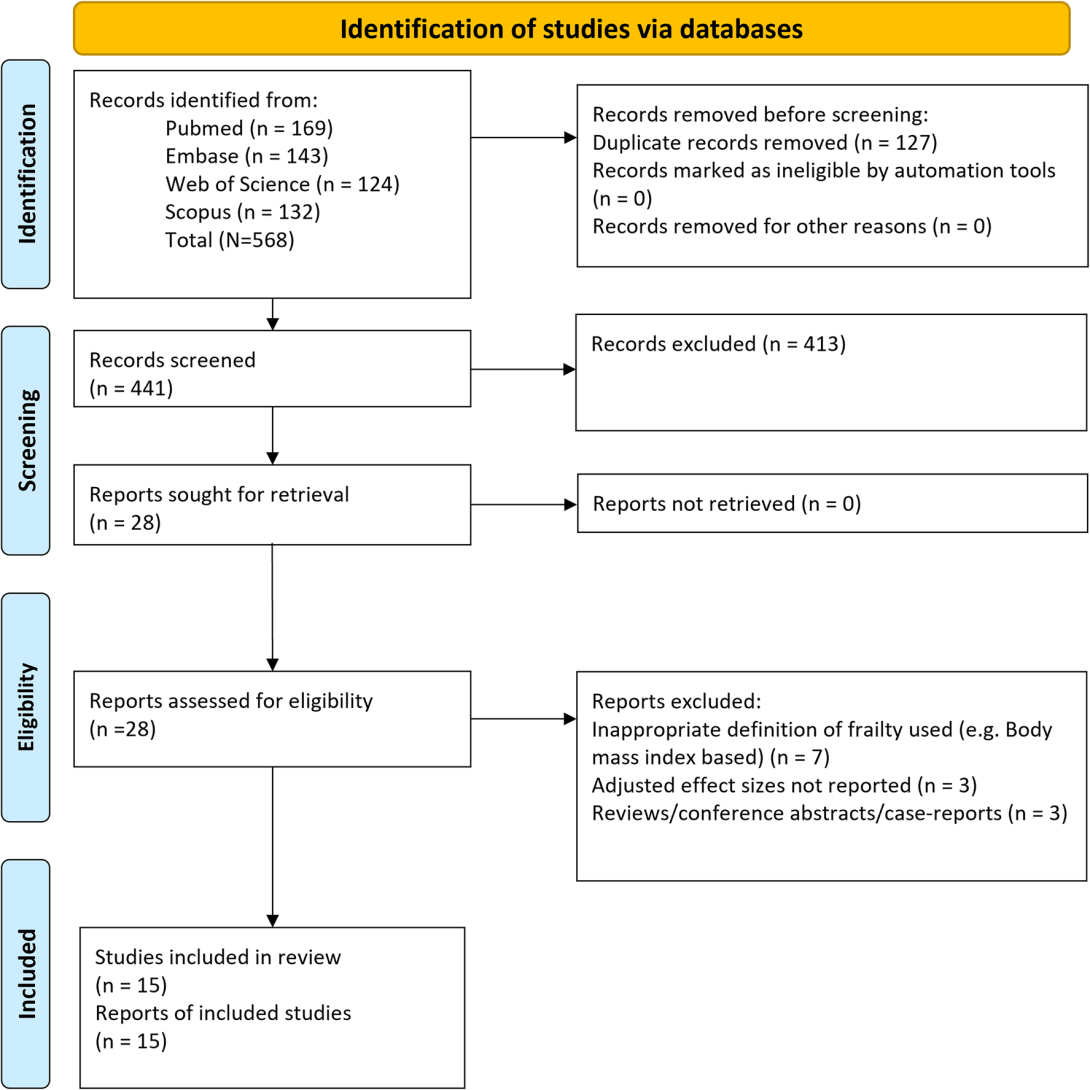
PRISMA flow chart showing the process of selection of studies

A total of 568 studies were identified by the initial search across the databases. Of them, 127 studies were removed as duplicates (Fig. 1). The titles and abstracts of 441 studies were screened, leading to exclusion of 413 papers. Full texts evaluation of the remaining 28 studies led to removal of 13 studies. Finally, 15 studies were eligible for inclusion [17–31] (Fig. 1).

### Characteristics of the included studies

Of the 15 included studies, six were prospective, seven retrospective, and two cross-sectional (Table 1). The main contributors were the United States (*n* = 5), Poland (*n* = 3), and Japan (*n* = 2). The mean age of patients ranged from 60 to 85 years, with males comprising a larger portion of the sample. Most studies had a long follow-up period, spanning from 12 to 60 months. The definitions and cut-offs for frailty showed significant variability among the studies. The selected studies included a total of 398,163 participants. Quality of the studies was satisfactory, with an average score of 7.13 on the NOS. Table 1 shows individual study scores.

**Table 1:** The included studies with their summarized characteristics

Author name, online year	Study design; location	Age, sex distribution	Follow up duration	Assessment of frailty	Sample size	Key outcomes assessed	Adjustment done for	NOS score
[17]	PC; Russia	Mean age 59 years Male (74%)	Mean follow up 49 months	Frailty index calculated using 31 parameters (the ability to perform daily activities, clinical status, laboratory markers, comorbidities) Low risk of frailty (< 0.375) High risk of frailty (≥ 0.375)	77	Mortality Response to CRT	Adjusted for age, gender, rhythm, NYHA class, left ventricular ejection fraction, left bundle branch block, and QRS width	7
[18]	RC; Hungary	Mean age 69 years Male (75%)	Median follow up 4.8 years	30-item frailty index; items were derived from medical history, other patient characteristics, and laboratory results Low risk of frailty (FI ≤ 0.210) High risk of frailty (FI > 0.210)	1004	Mortality	Mentioned that multivariable Cox proportional hazards models were used. However, variables adjusted in the models were not reported	7
[19]	RC; United States	Mean age 59 years Male (77%)	Median follow up 3.4 years	Baseline frailty estimated using the Rockwood Frailty Index (FI) Participants stratified into high (FI > 0.41) and low (FI ≤ 0.41) frailty groups	1676	Mortality Readmission	Adjusted for age, sex, race, heart failure aetiology (ischemic vs non-ischaemic), and NYHA functional class (II vs III)	8
[20]	RC; United States	Mean age 75 years Male (55%)	Outcomes assessed during the duration of hospital admission	Hospital Frailty Risk Score (HFRS) Low risk of frailty (HFRS < 5) High risk of frailty (HFRS > 15)	7575	In-hospital mortality Complication	Adjusted for age, sex, race, type of admission, bed size of hospital, location of hospital, primary expected payer, and Charlson Comorbidity Index	8
[21]	RC; Japan	Median age 85 years Male (42%)	Median follow up 4.1 years	Frailty assessed on the basis of impairments in 3 domains (walking, cognition, and activities of daily living). Scores calculated by summing across the 3 frailty variables Normal/no frailty (score 0); mild frailty (score of 1 or 2); severe frailty (score of 3 to 6)	103	Decompensated heart failure	Adjusted for age, sex, diabetes, hypertension, left ventricular ejection fraction, atrial fibrillation, chronic kidney disease, ischemic heart disease	8
[22]	PC; France	Mean age 78 years Male (75%)	Follow up 60 months	Frailty defined as a score of < 14 out of 17 points using the ONCODAGE G8 score	129	Mortality	Mentioned that adjustment for covariates with potential prognostic impact, including New York Heart Association class was done	7
[23]	RC; Japan	Mean age 76 years Male (63%)	Follow up of 12 months	Modified frailty index (mFI) used; based on the accumulation of 11 physiological Defects Low risk of frailty (mFI ≤ 2) High risk of frailty (mFI ≥ 3)	101	Readmission	Adjusted for sex, age, cardiac function, renal function, anemia, hyponatremia, ischemia, type of CIED, and new implantation	7
[24]	PC; United States	Mean age 81 years Male (71%)	Median follow up 4.6 years	Frailty scores calculated as an accumulation of 36 deficits Fit (score 0–9); mild frailty (score 10–12); moderate frailty (score 13–15); severe frailty (score ≥ 16)	469	Mortality	Adjusted for age, sex, left ventricular ejection fraction, QRS duration, kidney function, and type of implanted CRT device	6
[25]	CS; Poland	Median age 71 years Male (82%)	Follow up of 12 months	The Tilburg Frailty Indicator which is a 15-item tool A score of 5 or more points was considered as frailty	103	Complication	Adjusted for age, gender, body mass index	7
[26]	RC; United States	Mean age 63 years Male (60%)	Follow up of 12 months	Modified frailty index (mFI) used; based on the accumulation of 11 physiological Defects Low risk of frailty (mFI ≤ 2) High risk of frailty (mFI ≥ 3)	283	Mortality Readmission Complication	Adjusted for age, NYHA class, and type of cardiomyopathy	8
[27]	PC; Poland	Mean age 73 years Male (77%)	Follow up 12 months	The Tilburg Frailty Indicator which is a 15-item tool A score of 5 or more points was considered as frailty	246	Response to CRT	Variables used in regression model has not been reported. However, the reported effect size is from logistic regression model	6
[28]	RC; United States	Those with high frailty score were older and less likely to be male	Outcomes assessed during the duration of hospital admission	Hospital Frailty Risk Score Low-risk (score < 5) Intermediate-risk (Score 5–15) and high-risk (score > 15)	386,062	In-hospital Mortality Complications	Adjusted for a number of variables such as age, sex, weekend admission, median household income, dyslipidemia, smoking status, previous acute myocardial infarction/coronary artery bypass grafting/ ischemic heart disease	7
[29]	CS; Poland	Mean age 74 years Male (82%)	Mean follow up of 654 days	The Tilburg Frailty Indicator which is a 15-item tool A score of 5 or more points was considered as frailty	156	Decompensated heart failure Readmission	Adjusted for age, hypertension, dyslipidemia, diabetes and ejection fraction	7
[30]	PC; Italy	Mean age 63 years Male (74%)	Follow up 24 months	Based on allostatic overload: poor baseline psychological and psychosomatic profile	117	Mortality	Psychological mean scores and psychological diagnoses (depression/anxiety, etc.) were tested in univariate Cox regression analysis. Those variables significantly related to post-ICD complications in univariate analysis were also entered into the multivariate Cox regression analysis, adjusted for sex and age	7
[31]	PC; Spain	Mean age 73 years Male (53%)	Follow up 12 months	Assessed using Fried and Walston definition; includes five elements i.e., unintended weight loss, exhaustion, physical activity, time to walk, and grip strength, with frailty defined as meeting three or more core elements	102	Decompensated heart failure	Adjusted for dyslipidemia, hypertension, diabetes, and minute ventilation/carbon dioxide production slope	7

*RC* retrospective cohort, *CRT* cardiac resynchronization therapy, *PC* prospective cohort, *CS* cross sectional, *CIED* cardiac implantable electrical devices, *NYHA* new york heart association class, *ICD* implantable cardioverter defibrillator

### Narrative synthesis of the findings of the included studies

The included studies indicated a significant association between frailty and adverse outcomes. For instance, frail individuals experienced a 4- to 9 fold increased risk of all-cause mortality, with one study reporting a staggering 87.5% survival rate for non-frail patients compared to only 47.2% for frail patients over a 10 year follow-up [17]. Additionally, frailty was documented to significantly impact the response to CRT, with frail patients showing less improvement in left ventricular ejection fraction and higher rates of non-response, evidenced by a study that identified frailty as an independent predictor of response to CRT (odds ratio: 0.81) [27]. Moreover, frail patients exhibited higher rates of complications and readmissions compared to their robust counterparts. The increased frailty levels not only correlated with worse clinical outcomes but also led to higher healthcare costs and longer hospital stays, emphasizing the urgent need for frailty assessment in clinical practice.

## Meta-analysis based specific findings

### Short-term outcomes

**Fig. 2:**
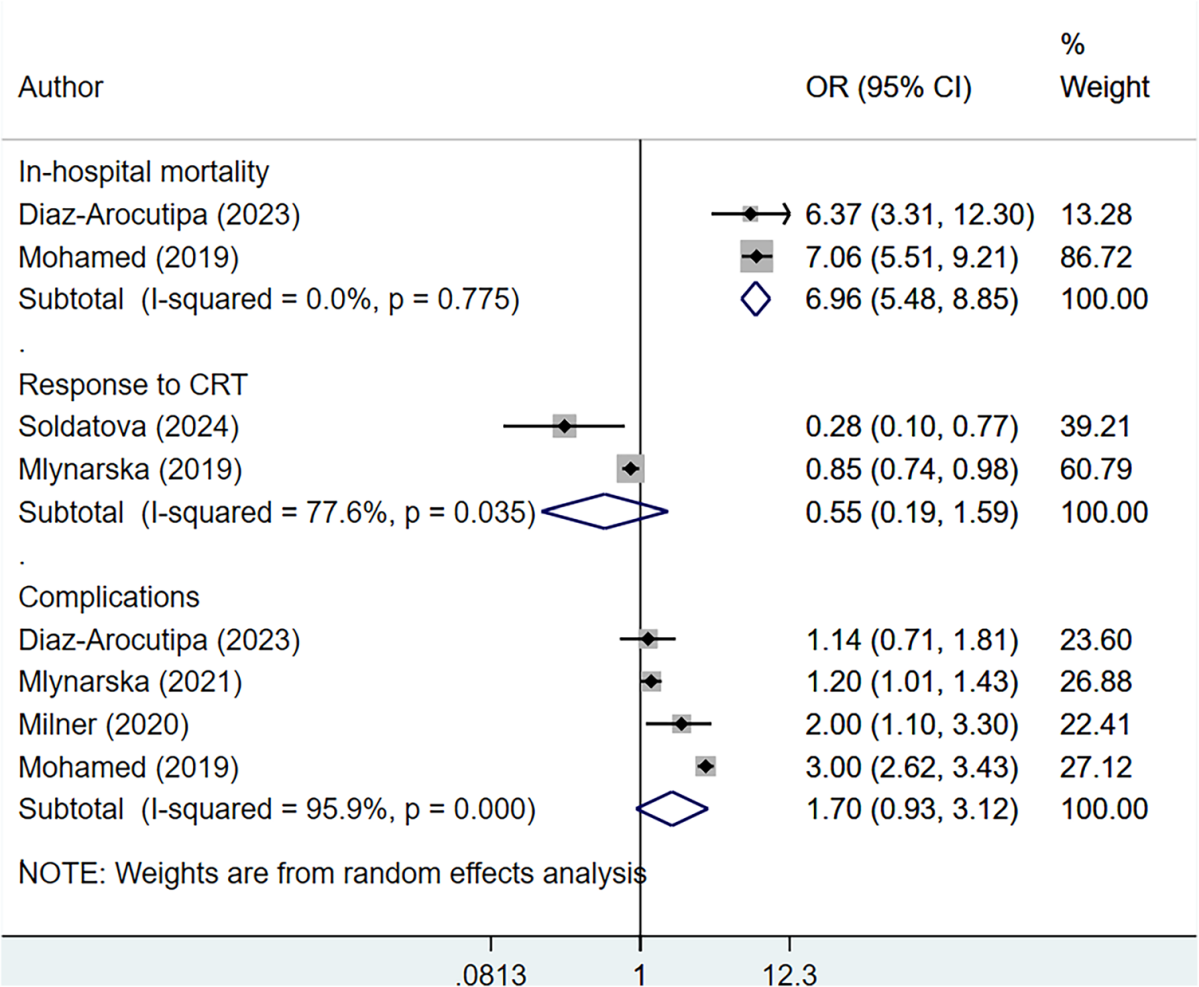
Risk of in-hospital mortality, complications and response to CRT among frail participants undergoing cardiac resynchronization therapy, compared to non-frail participants

Frailty was associated with an increased risk of in-hospital mortality (OR 6.96, 95% CI 5.48, 8.85; *n* = 2, *I*^2^ = 0.0%) (Fig. 2). However, the major weight in the analysis was allocated to the study by Mohamed (2019) et al. because of the higher sample size (Fig. 2) [28]. Even after removing of this study from the analysis, the risk of in-hospital mortality was high (OR 6.37, 95% CI 3.31, 12.3); however, it was based on only one study by Diaz-Arocutipa (2023) et al. [20]. The effect of frailty on response to CRT was not statistically significant (OR 0.55, 95% CI 0.19, 1.59; *n* = 2, *I*^2^ = 77.6%) (Fig. 2). When sensitivity analysis was performed including only studies that utilized the frailty index, only one study (i.e., Soldatova et al.) reported on the response to CRT, with an OR of 0.28 (95% CI 0.10, 0.77) [17]. The risk of complications was comparable in frail and non-frail patients (OR 1.70, 95% CI 0.93, 3.12; *n* = 4, *I*^2^ = 95.9%) (Fig. 2). However, the direction of the pooled effect size suggests around 1.5-times higher risk of complications in frail participants (Fig. 2). When sensitivity analysis was conducted using only studies that employed the frailty index, Milner et al. was the only study that reported on the risk of complications, showing an OR of 2.00 (95% CI 1.10, 3.30) [26]. The complications reported in the included studies were those related to vascular, pericardial, pneumothorax, infectious, device retrieval/replacement, defibrillator cardioverter electric shock, and major adverse cardiovascular events.

### Outcomes on long-term follow-up

**Fig. 3:**
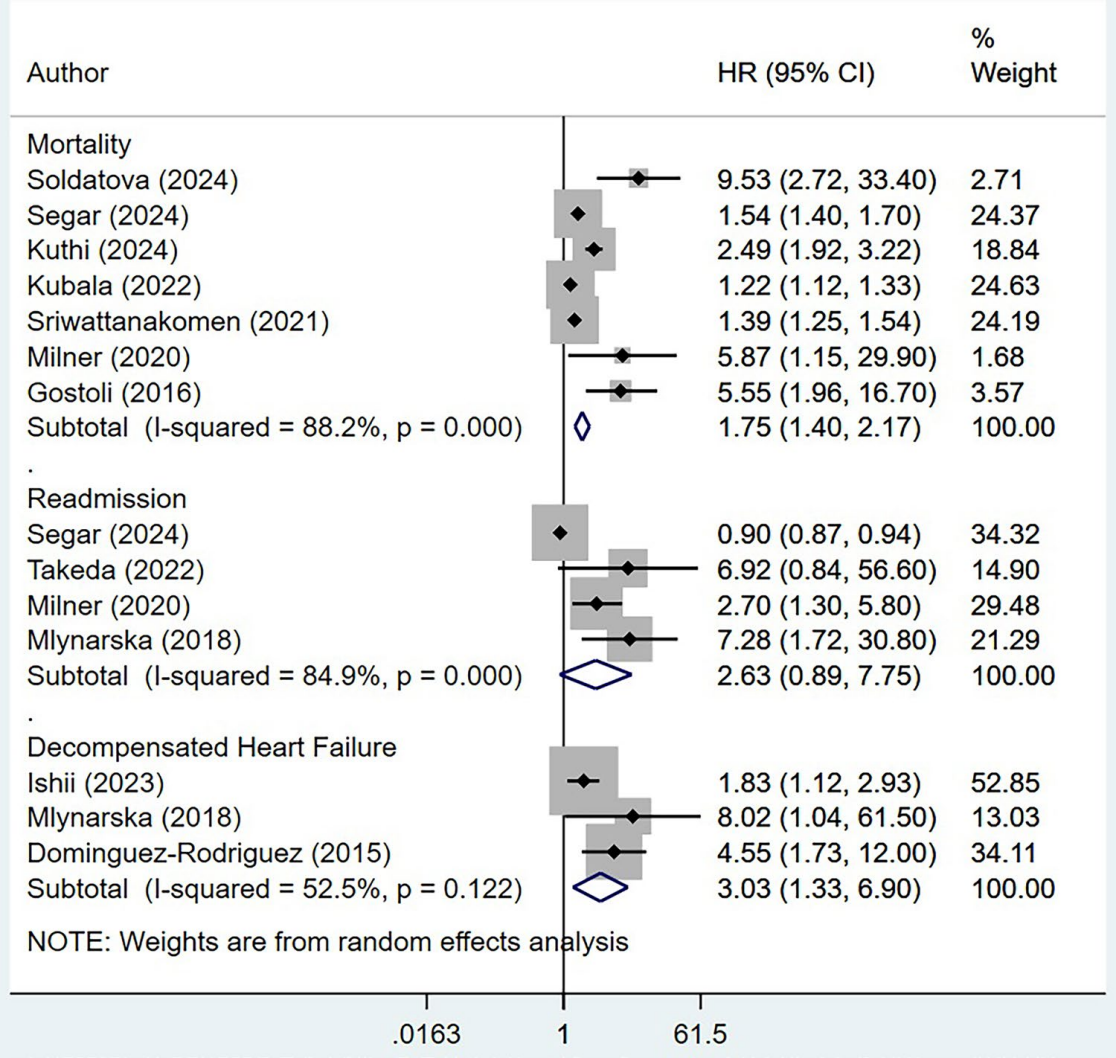
Risk of long-term mortality, decompensated heart failure and readmission among frail participants undergoing cardiac resynchronization therapy, compared to non-frail participants

The risk of mortality on long-term follow up was significantly higher in frail patients (HR 1.75, 95% CI 1.40, 2.17; *n* = 7, *I*^2^ = 88.2%), compared to patients without frailty (Fig. 3). Sensitivity analysis with studies using Frailty index showed a comparatively stronger and significant association of frailty with risk of mortality (HR 2.59, 95% CI 1.53, 4.38; *n* = 4, *I*^2^ = 86.1%) (Supplementary Fig. 1). Similarly, the risk of decompensated heart failure was higher in frail participants (HR 3.03, 95% CI 1.33, 6.90; *n* = 3, *I*^2^ = 52.5%). While the risk of readmission appeared to be higher in frail patients, however, it did not achieve statistical significance (HR 2.63, 95% CI 0.89, 7.75; *n* = 4, *I*^2^ = 84.9%) (Fig. 3). Similar findings were observed in sensitivity analysis with studies using frailty index (HR 1.90, 95% CI 0.66, 5.50; *n* = 3, *I*^2^ = 83.1%) (Supplementary Fig. 2).

### Publication bias

Publication bias assessment did not find evidence of publication bias for the risk of mortality and decompensated heart failure (Egger’s *p* > 0.05). However, possible publication bias was found for the risk of readmission, as indicted by the Egger’s *p*-value of 0.02. Funnel plots for each of the three outcomes are shown in Supplementary Figs. 3, 4, 5.

## Discussion

The findings of this review underscore the significant impact of frailty on outcomes following CRT. Frailty was associated with substantially higher odds of in-hospital mortality. While the effect of frailty on the response to CRT did not reach statistical significance, there was a trend towards reduced response rates among frail patients. Additionally, our analysis revealed a notable trend towards an increased risk of complications in frail patients. The direction of the pooled effect size suggests that frail patients may be at approximately 1.5-times higher risk of experiencing complications compared to their non-frail counterparts. On long-term follow-up, frailty correlated with significantly higher risks of mortality and decompensated heart failure compared. While the risk of readmission appeared to be elevated in frail participants, the association did not achieve statistical significance. Overall, the findings of this review highlight the critical role of frailty assessment in risk stratification and prognostication among patients undergoing CRT.

Frailty is a multifaceted syndrome characterized by diminished physiologic reserves and increased vulnerability to stressors [6, 7]. Our results showed the association of frailty with the increase rates of adverse outcomes in patients after CRT. We think that this is due to the associated comorbidities rather than the efficacy of CRT itself. Frail patients often present with multiple comorbidities such as cardiovascular disease, diabetes, and chronic kidney disease, which can exacerbate the complexity of managing heart failure and increase the risk of adverse events post-CRT [32–35]. Moreover, frailty is closely linked with age-related physiological decline, including sarcopenia, cognitive impairment, and dysregulation of inflammatory pathways [36–39]. These age-related changes may impair the myocardial response to CRT, diminish the efficacy of cardiac resynchronization, and predispose frail individuals to persistent heart failure symptoms and post-CRT decompensation [36–38]. Frailty can impair physical function and mobility, limiting participation in rehabilitation and adherence to management guidelines, which may worsen outcomes [39–41]. It is also linked to neuroendocrine and immune alterations, leading to inflammation, oxidative stress, and autonomic dysregulation [42–45]. These factors could contribute to cardiac remodeling and deterioration post-CRT [45, 46]. Addressing the complex interplay of these issues is crucial for improving outcomes and quality of life for frail CRT patients.

It is important to note that the decision to implant CRT devices in frail participants requires careful consideration of the balance between potential benefits and risks. While CRT has demonstrated efficacy in improving cardiac function, the presence of frailty may attenuate these benefits. The adverse outcomes observed in frail participants post-CRT are likely more attributable to the underlying frailty syndrome and comorbidities rather than the efficacy of CRT itself. Frailty encompasses a range of physiological impairments such as sarcopenia, reduced immune function, and comorbid conditions, which could predispose to complications and mortality regardless of the intervention. However, the increased risk associated with CRT in frail participants does not automatically preclude its use in these participants. They may still benefit from CRT, especially if they have significant heart failure symptoms and reduced quality of life that outweigh the risks. Clinicians could identify frail patients who may derive the most benefit, such as those with moderate frailty but with good cognitive function and social support, allowing for appropriate management and monitoring post-procedure [47]. Implementing individualized care plans that address the specific needs and risks associated with frailty seems important. This includes weighing the potential benefits of CRT against the risks for each patient, considering their overall health status, comorbidities, and functional capabilities [47]. Routine frailty assessments should be integrated into the pre-CRT evaluation process. Frailty scores can guide discussions with patients regarding the expected outcomes and potential complications of CRT [48]. To enhance the safety of CRT in frail patients, it is essential to adopt a multidisciplinary management approach [49]. This may involve optimizing medical therapy prior to CRT, ensuring that patients are stable before undergoing the procedure, and providing extensive postoperative support, including rehabilitation and monitoring for complications. Establishing a collaborative framework between geriatricians and cardiologists is critical for improving outcomes in frail patients [49]. Regular case discussions and joint clinics can facilitate better communication and decision-making. Cardiologists could prioritize comprehensive pre-procedural assessments that include frailty evaluations. Geriatricians can provide expertise in assessing frailty, identifying comorbidities, and optimizing perioperative care. The training programs focused on the complexities of managing frailty in heart failure patients can enhance understanding and collaboration between the two specialties.

The strength of our review lies in its comprehensive and systematic approach. This is perhaps the most updated review that objectively assesses the association between frailty and adverse outcomes following CRT. There are some important limitations of this review which warrants consideration. One of the significant limitations is the inherent heterogeneity among included papers, possibly due to differences in patient populations, frailty assessments, and outcome measures. This high heterogeneity may limit the generalizability of our findings. It is important to note that the underlying conditions such as hypertension, atherosclerosis, cardiomyopathy, malnutrition, sarcopenia, type 2 diabetes, and malignancy are likely to be associated with a higher risk of complications and increased mortality rates and these should be accounted for in the analytic model while assessing the relationship between frailty and outcomes of CRT. Although we included studies that reported confounder- or covariate-adjusted effect sizes, there remains the possibility that some important variables or confounders were not accounted for, which could potentially alter the true strength of the association.

Methodological variability, including differences in frailty assessment methods, cut-offs used to define frailty and outcome definitions, may introduce bias and confound the interpretation of results. The lack of standardization in frailty assessment methods and definitions across studies may affect comparability and consistency of findings. Additionally, potential publication bias was found for the “readmission” outcome. Higher representation of cross-sectional and retrospective data limits the ability to establish causality or assess temporal relationships between frailty and CRT outcomes.

More prospective longitudinal studies are needed to clarify temporal relationship between frailty and CRT outcomes. Additionally, further investigation is needed to understand the underlying mechanisms linking frailty to adverse outcomes following CRT, including the role of inflammation, neurohormonal dysregulation, and altered pharmacokinetics. Further, there is a need to standardize frailty assessment methods and definitions across studies to facilitate comparability of data.

## Supplementary Information

Below is the link to the electronic supplementary material.

Supplementary file 1 Sensitivity analysis using studies employing frailty index and examining the risk of long-term mortality among frail participants, compared to non-frail participants (TIF 3171 KB)

Supplementary file 2 Sensitivity analysis using studies employing frailty index and examining the risk of readmission among frail participants, compared to non-frail participants (TIF 3265 KB)

Supplementary file 3 Funnel plot for publication bias related to risk of mortality (TIF 3148 KB)

Supplementary file 4 Funnel plot for publication bias related to risk of decompensated heart failure (TIF 3148 KB)

Supplementary file 5 Funnel plot for publication bias related to risk of readmission (TIF 3148 KB)

## Data Availability

The data that support the findings of this study are available from the corresponding author upon reasonable request.
