# Supplementary figures and images for: Association between frailty and adverse outcomes after cardiac resynchronization therapy: a systematic review and meta-analysis
Source: Eur Geriatr Med. 2024 Dec 4;16(1):165–77. doi: 10.1007/s41999-024-01112-9 (PMC11850548; doi:10.1007/s41999-024-01112-9)

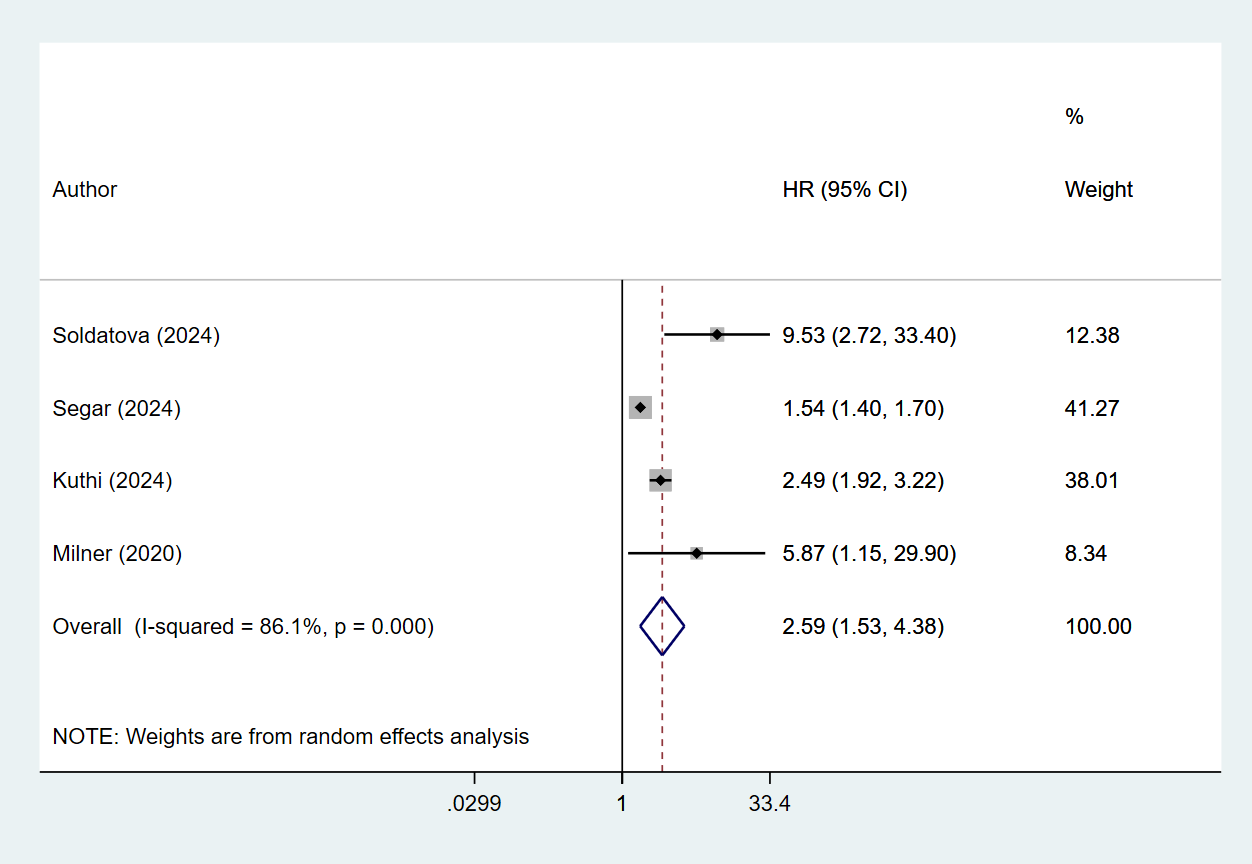

Supplement: Supplementary file 1 — Sensitivity analysis using studies employing frailty index and examining the risk of long-term mortality among frail participants, compared to non-frail participants (TIF 3171 KB) [file 41999_2024_1112_MOESM1_ESM.tif]

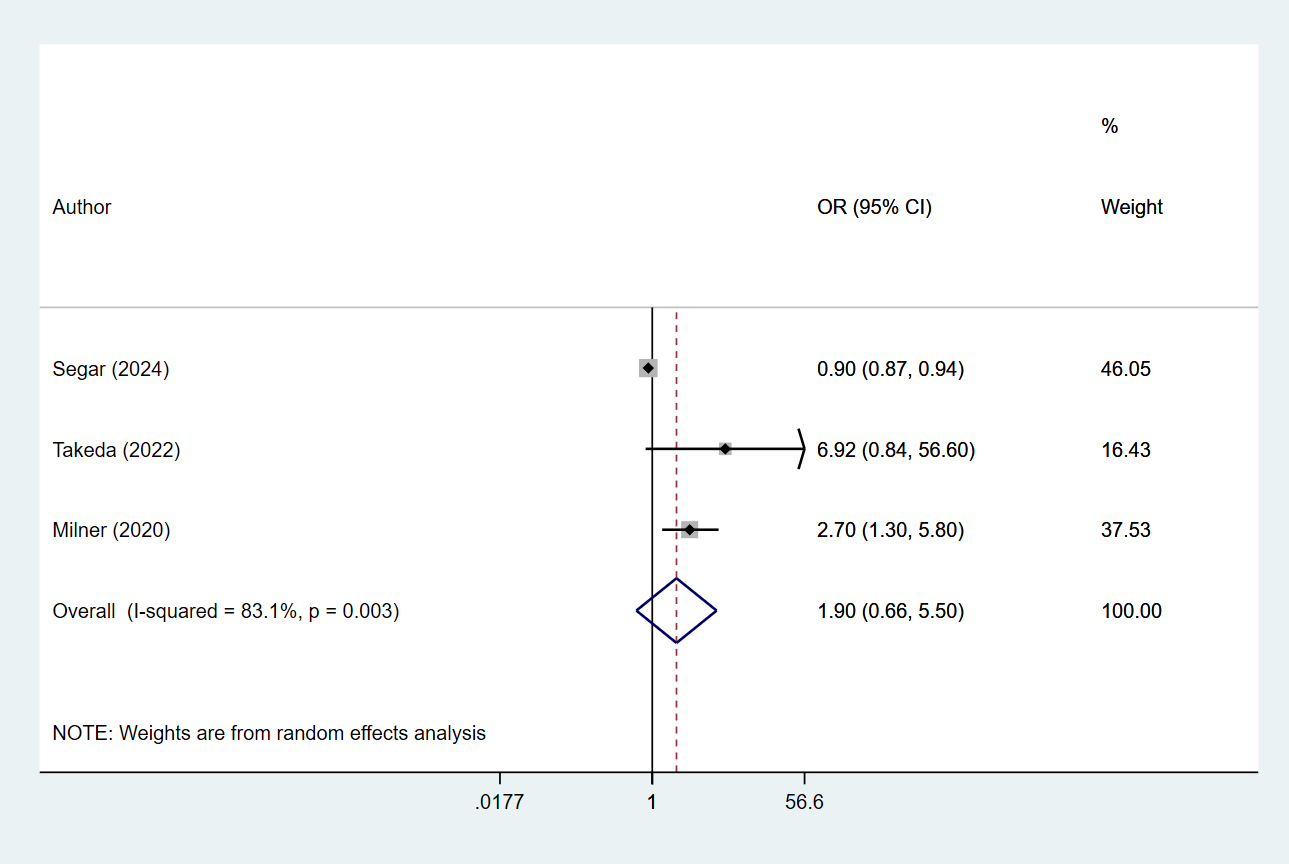

Supplement: Supplementary file 2 — Sensitivity analysis using studies employing frailty index and examining the risk of readmission among frail participants, compared to non-frail participants (TIF 3265 KB) [file 41999_2024_1112_MOESM2_ESM.tif]

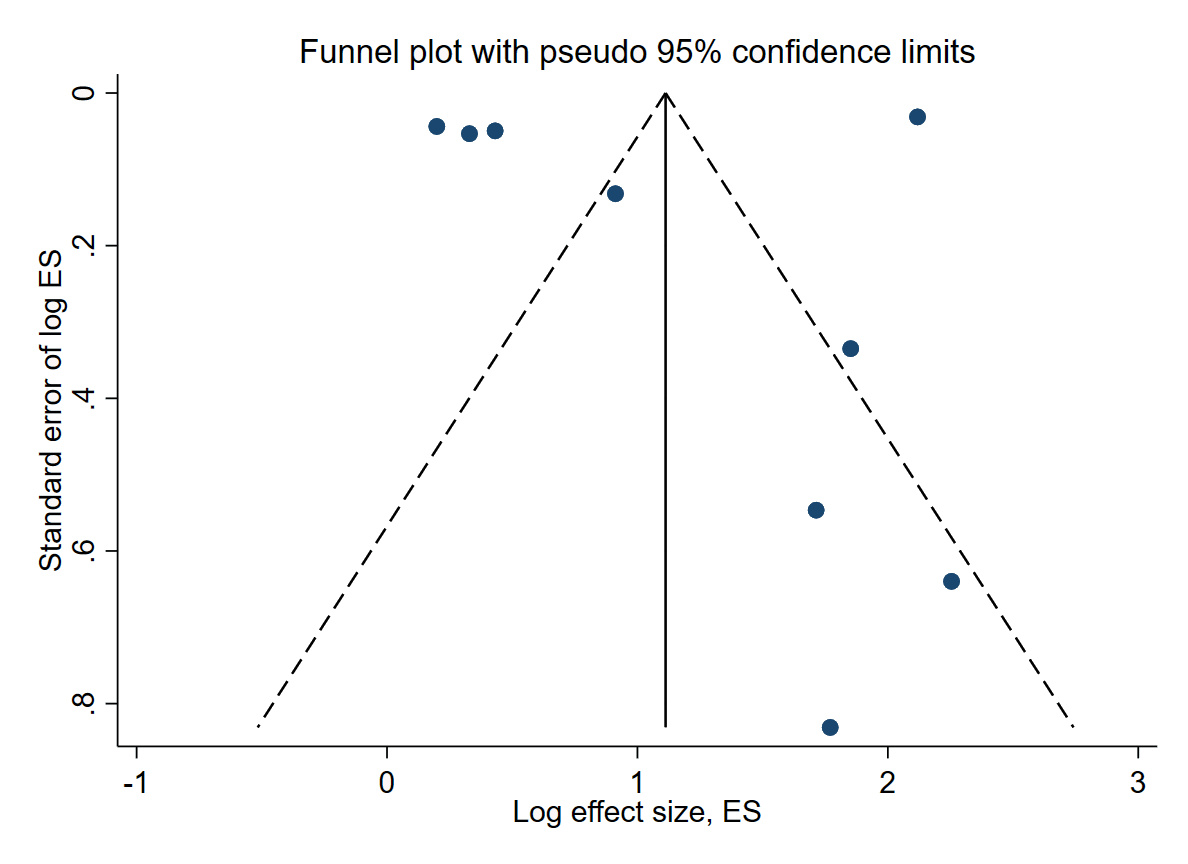

Supplement: Supplementary file 3 — Funnel plot for publication bias related to risk of mortality (TIF 3148 KB) [file 41999_2024_1112_MOESM3_ESM.tif]

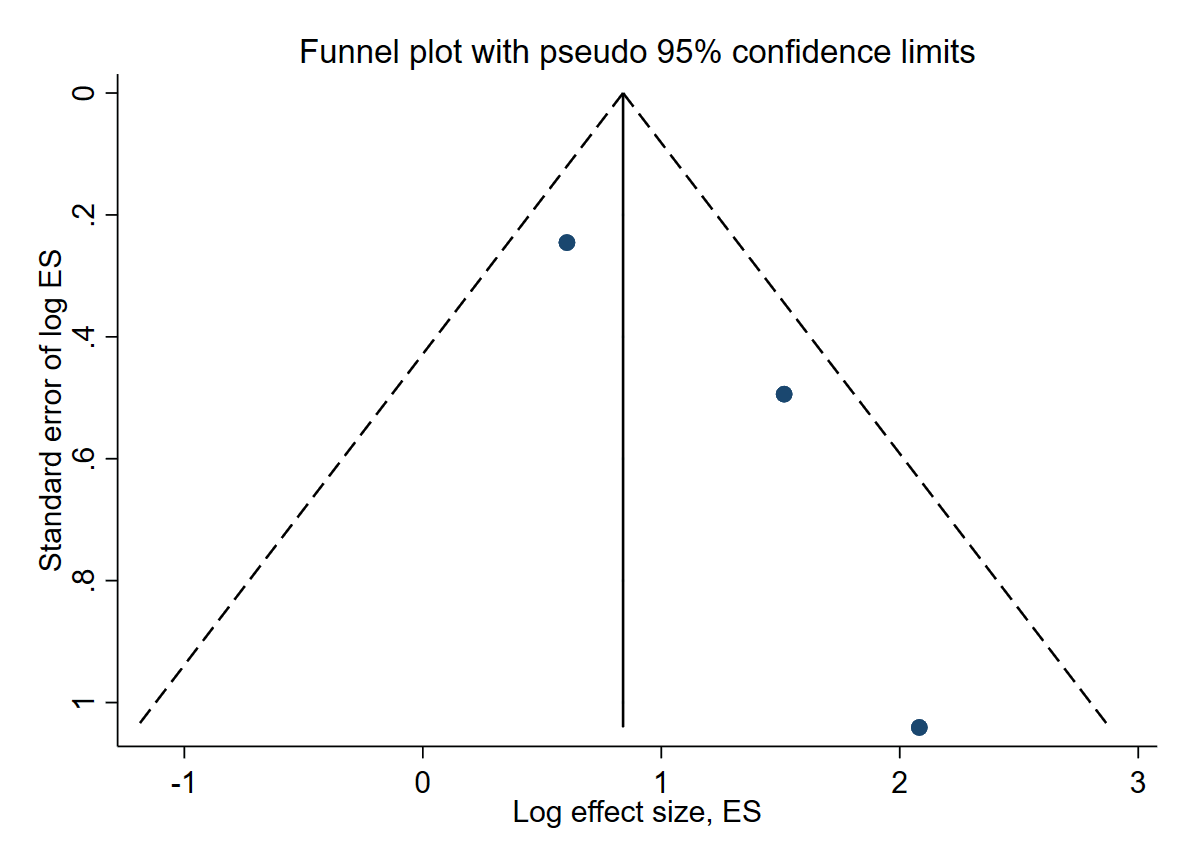

Supplement: Supplementary file 4 — Funnel plot for publication bias related to risk of decompensated heart failure (TIF 3148 KB) [file 41999_2024_1112_MOESM4_ESM.tif]

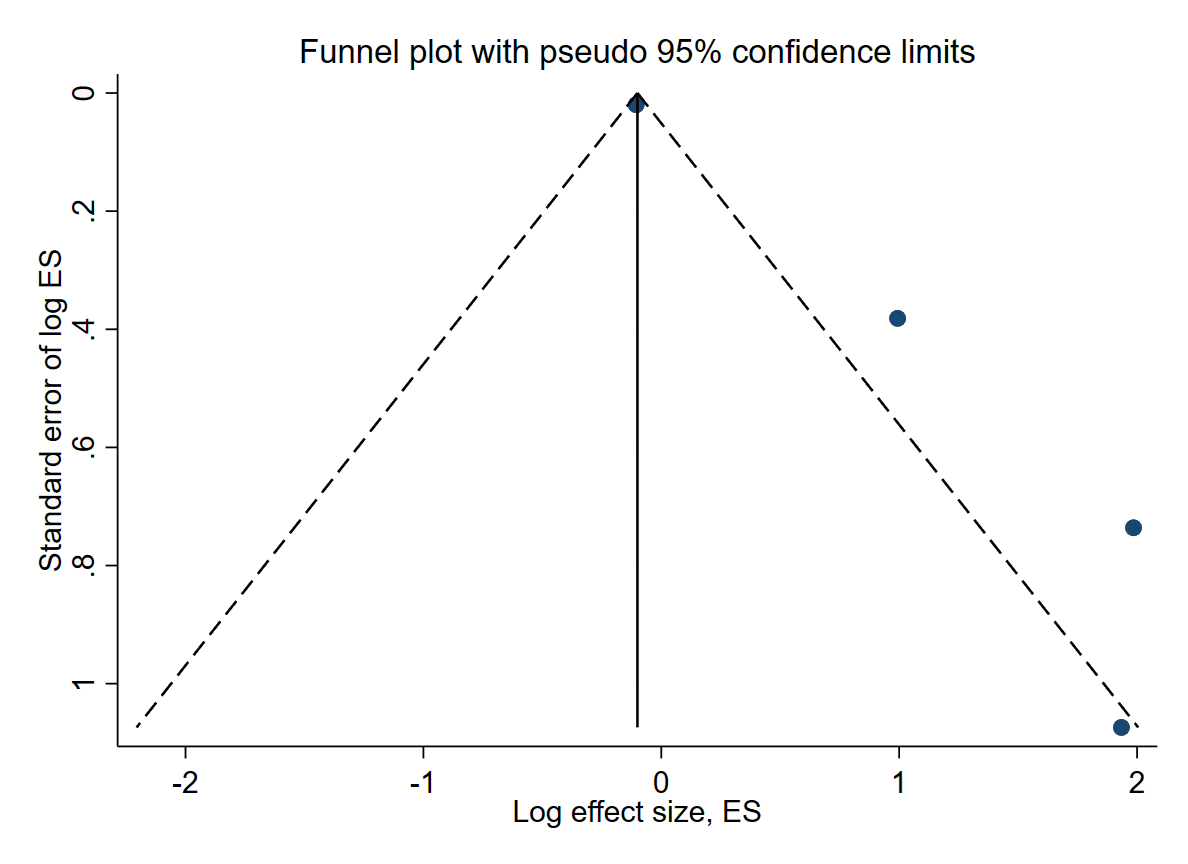

Supplement: Supplementary file 5 — Funnel plot for publication bias related to risk of readmission (TIF 3148 KB) [file 41999_2024_1112_MOESM5_ESM.tif]
